# Effect of pre- and post-treatment α-fetoprotein levels and tumor size on survival of patients with hepatocellular carcinoma treated by resection, transarterial chemoembolization or radiofrequency ablation: a retrospective study

**DOI:** 10.1186/1471-2482-14-40

**Published:** 2014-07-04

**Authors:** Adriana Toro, Annalisa Ardiri, Maurizio Mannino, Maria Concetta Arcerito, Giovanni Mannino, Filippo Palermo, Gaetano Bertino, Isidoro Di Carlo

**Affiliations:** 1 Department of Surgical Sciences, Organ Transplantation and Advanced Technologies, University of Catania, Cannizzaro Hospital, Via Messina, 829, Catania 95126, Italy; 2 Department of Internal Medicine and Systemic Disease, Hepatology Unit, University of Catania, S. Marta Hospital, Catania, Italy; 3 University of Catania, Catania, Italy; 4 Infectious Diseases Unit, Cannizzaro Hospital, Catania, Italy; 5 Department of Internal and Specialist Medicine, Section of Infectious Disease, University of Catania, Garibaldi Hospital, Catania, Italy; 6 Department of Surgery, Hamad Medical Corporation, Doha, Qatar

**Keywords:** Hepatocellular carcinoma, Hepatic resection, Transarterial chemoembolization, Radiofrequency ablation, Alpha-fetoprotein, Tumor size, Survival

## Abstract

**Background:**

We evaluated treatment modalities and survival in patients with hepatocellular carcinoma (HCC), by pre-treatment and 3-month post-treatment serum alpha-fetoprotein (AFP) levels and pre-treatment tumor diameters.

**Methods:**

We retrospectively reviewed 57 patients treated for HCC in our department from January 2002 to December 2012, including their sex, type of hepatitis, Child class, pre-treatment tumor size, pre-treatment levels of albumin, aspartate aminotransferase (AST), alanine aminotransferase (ALT), gamma-glutamyltransferase (GGT), red blood cells, hemoglobin, and total bilirubin, pre- and 3-month post-treatment serum AFP, and treatment modality (transarterial chemoembolization, resection or radiofrequency ablation). Survival was analyzed at 1, 3, and 5 years after treatment.

**Results:**

The 57 patients included 44 men and 13 women, of whom 44 had hepatitis C virus (HCV) infection, 3 had hepatitis B virus (HBV) infection, 3 had both HBV and HCV infection, 1 had both HBV and hepatitis D virus infection, and 3 had alcohol-related liver cirrhosis. Both pre- and post-treatment serum AFP levels significantly correlated with recurrent tumor size (*P* < 0.05 for both). Pre-treatment tumor size did not correlate with recurrent tumor size. Patients who underwent hepatic resection survived significantly longer than those who underwent transarterial chemoembolization or radiofrequency ablation (*P* < 0.05).

**Conclusions:**

Serum AFP level is useful in diagnosing tumor recurrence and predicting prognosis in HCC patients treated by hepatic resection, transarterial chemoembolization, and radiofrequency ablation. Hepatic resection remains the treatment of choice for HCC in suitable patients.

## Background

Most primary liver cancers are hepatocellular carcinoma (HCC), which is the third most common source of cancer fatalities worldwide. The main etiologies of HCC are infection of the liver by hepatitis B virus (HBV) or hepatitis C virus (HCV), and alcohol abuse [1].

Alpha-fetoprotein (AFP) is an oncofetal protein produced by fetal hepatocytes, yolk-sac cells, and normal gastrointestinal cells immediately after birth. Serum AFP level decreases gradually after birth to <10 ng/mL within 300 days [2,3]. Normal adult serum AFP level is <20 ng/mL [2]. Serum AFP level may be high in patients with drug or alcohol abuse or with chronic liver disease such as hepatitis or cirrhosis, but in these cases, the level is usually <100 ng/mL [4]. The Italian guidelines and the American Association for the Study of Liver Diseases guidelines consider serum AFP >200 ng/mL to be diagnostic for HCC [2]. Some studies have suggested using a serum AFP level of >400 ng/mL to diagnose HCC [5], or a solid mass >2 cm in diameter with typical features of HCC on at least one imaging study in a patient with liver cirrhosis [2].

High serum AFP levels occur in 60–70% of HCC patients; however, serum AFP levels remain in the normal range in 15–30% of HCC patients [5]. AFP plays an important role in the regulation of both oncogenic and ontogenetic growth [6]. Although early studies indicated that AFP and its derived peptide fragments can inhibit oncogenic growth [7], more recent studies have shown that AFP can promote HCC cell growth. AFP^+^ HCC has higher cell proliferative activity than AFP^-^ HCC, as measured by the Ki-67 index [8]. Down-regulation of AFP can suppress HCC cell growth [9]. High serum AFP level correlates with more aggressive behavior and poorer prognosis of HCC [2,10].

Serum AFP level has been suggested to directly reflect tumor size, and the ratio of serum AFP level to tumor diameter to predict recurrence after curative resection is better than serum AFP alone [10]. To the best of our knowledge, no reported studies evaluated whether the relationship between pre- and post-treatment serum AFP levels predict recurrent tumor size in HCC patients treated by hepatic resection (HR), transarterial chemoembolization (TACE), or radiofrequency ablation (RFA).

We evaluate relationships between treatment modality (HR, TACE, and RFA) and survival in HCC patients, according to pre-treatment and 3-month post-treatment serum AFP levels and pre-treatment tumor diameter. We also evaluated the relationships between 3-month post-treatment serum AFP levels and recurrent tumor size.

## Methods

This retrospective study was approved by the local ethics committee of Cannizzaro Hospital, Catania, Italy.

From the patients who were treated for HCC in our department from January 2002 to December 2012, we included those aged > 18 years, with Child class A or B HCC. Diagnosis of HCC and measurement of tumor diameter were based on computed tomography (CT) or magnetic resonance imaging findings. Data collected included sex, age, type of hepatitis, Child class, tumor size, pre-treatment tumor size, levels of albumin, aspartate aminotransferase (AST), alanine aminotransferase (ALT), gamma-glutamyltransferase (GGT), red blood cells, hemoglobin, and total bilirubin, pre- and post-treatment serum AFP levels, and treatment modality. Survival was analyzed at 1, 3, and 5 years after treatment. Tumor size was defined as maximum tumor diameter. If multiple tumors were present, tumor size was defined as the sum of the maximum diameters of all tumors.

All patients were followed up, with measurements of serum AFP and CTs, every 3 months for the first year after treatment, and then every 6 months for the next 4 years.

Patients were divided by their treatment modalities into the HR group, the TACE group, and the RFA group.

Among patients who underwent HR, the selection criteria were Child class A disease, and indocyanine green retention rate at 15 min, as evaluated by the Makuuchi algorithm [11]. Thus some patients with small HCCs and negative results by the Makuuchi algorithm who requested large resections were shifted to RFA or TACE procedures. Resection types were defined according to the Brisbane classification [12]. Curative resection was defined as a 1-cm surgical margin of non-cancerous tissue on postoperative histological examination.

Among patients who underwent TACE, selection criteria were either Child class A or B disease with multiple tumors not suitable for surgery or RFA. The TACE procedure involved cannulation of the femoral artery using the Seldinger technique under local anesthesia. A total of 50 mg of epirubicin emulsified in iodized oil was used for chemotherapy; embolization was performed using gelatin sponge particles. CT was performed 3 weeks after treatment to assess the results. All patients with hyperdense images related to the treated tumors in basal CT scan were considered to have received effective treatments.

Among patients who underwent RFA, selection criteria were either Child class A or B disease with unique or multiple nodules suitable for this technique. RFA was performed under local anesthesia with sedation, using a 460-KHz generator. The electrode consisted of 9 hook-shaped prongs (StarBurst XL; RITA Medical Systems, Tyco Healthcare, Burlington, MA, USA), which can ablate a 5.0-cm diameter area. The entire tumor plus a 0.5–1.0-cm margin of surrounding normal hepatic tissue was ablated. In the first CT scan after the treatment, absence of arterial enhancement associate with necrosis of the tumor was considered to signify effective treatment.

Patients’ survival rates were analyzed and compared according to treatment modality, pre-treatment serum AFP level, 3-month post-treatment serum AFP level, and pre-treatment tumor size.

### Statistical analysis

Qualitative data were expressed as absolute frequency and relative frequency. Quantitative data were expressed as mean and standard deviation with minimum and maximum. Variables were calculated by Pearson’s *r* coefficient. Survival was calculated using the Kaplan–Meier method, and compared using the log-rank (Mantel–Cox) test. *P* < 0.05 was considered significant.

## Results

A total of 103 HCC patients were observed during the study period, of whom 46 patients were excluded (27 were in Child class C and 19 who had no indication for surgery refused to undergo RFA and TACE). Finally, 57 patients were included in the study, of whom 44 were men (77.2%) and 13 were women (22.8%), aged 40–86 years (mean age: 68.5 ± 9.81 years; mean age for men: 68.8 ± 9.25 years; mean age for women: 67.2 ± 9.13 years); 25 had Child Class A disease and 32 had class B disease; 44 patients (77.2%) had HCV infection, 3 (5.3%) had HBV infection, 3 (5.3%) had both HBV and HCV infection, and 1 (1.7%) had both HBV and hepatitis D virus infection. All patients with hepatitis virus infection had liver cirrhosis; 3 (5.3%) had alcohol-related cirrhosis, 1 (1.7%) had cryptogenic cirrhosis, and 2 (3.5%) had no cirrhosis. Twenty patients (35.1%) underwent HR, 27 (47.4%) TACE, and 10 (17.5%) RFA.

Pre-treatment laboratory levels were mean albumin: 2.13 ± 0.53 g/dL, range: 1.84–5.13 g/dL (normal range [NR]: 4.30–5.10 g/dL); mean AST: 77 ± 7.31 U/L, range: 17–217 U/L (NR: 8–39 U/L); mean ALT: 80.2 ± 10.3 U/L, range: 9–244 U/L (NR: 9–52 U/L); mean GGT: 123.8 ± 8.24 U/L, range: 23–857 U/L (NR: 9–50 U/L); mean prothrombin activity: 79.04 ± 5.37%, range: 43%–112% (NR: 70–140%); mean red cell count: 4.35 ± 1.23 M/L, range: 2.82–6.30 M/L (NR: 4.04–6.13 M/L); mean hemoglobin: 12.4 ± 3.27 g/dL, range: 6.9–17.10 g/dL (NR: 12.2–18.2 g/dL); mean total bilirubin: 1.13 ± 0.27 mg/dL, range: 0.01–2.76 mg/dL (NR: 0.2–1.3 mg/dL).

Mean pre-treatment serum AFP level was 211.52 ± 819.53 ng/mL, range: 1.30–6000.00 ng/mL (NR: 0–20 ng/mL). Mean tumor size was 34.67 ± 22.52 mm, range: 2.9–115 mm. Mean post-treatment serum AFP level was 70.50 ± 122.17 ng/mL, range: 2.1–546 ng/mL (NR: 0–20 ng/mL). Mean survival time was 24.63 ± 20.01 months (range: 1–120 months).

### Hepatic resection group

The HR group included 20 patients (35.1% of the total)—18 males (90.0%) and 2 females (10.0%)—with a mean age of 63.56 ± 9.87 years; all HR patients had Child class A disease.

**Figure 1:**
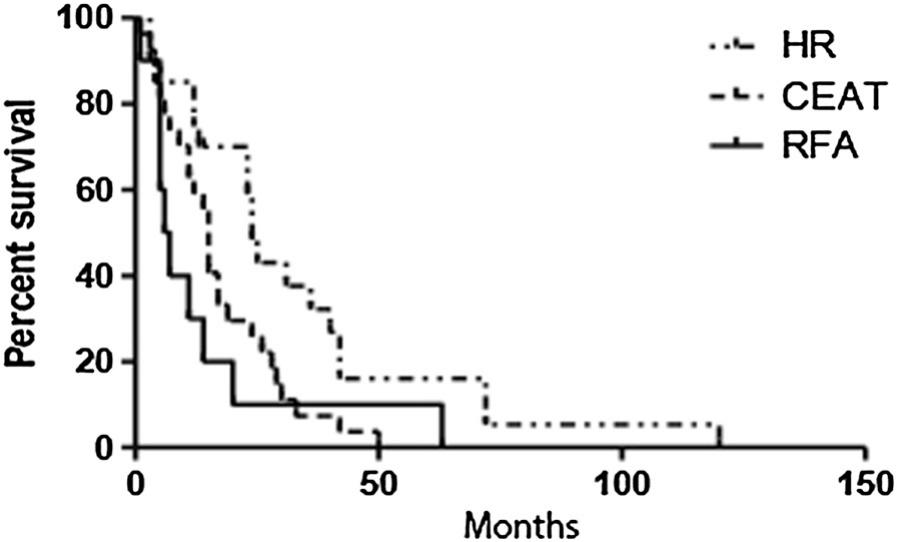
Survival of all patients after hepatic resection, transarterial chemoembolization, and radiofrequency ablation.

Their indocyanine green retention rates at 15 min were <10% in 11 patients (55%) and >10% in 9 patients (45%). Three patients (15%) underwent right hepatic resection, 7 (35%) underwent bisectionectomy, 6 (30%) underwent sectionectomy, and 4 (20%) underwent wedge resection. No perioperative morbidity was observed and no perioperative deaths occurred. All resections were curative. The mean postoperative hospital stay was 9 days. Two patients (10%) in this group underwent second hepatic resections because of tumor recurrence.In the HR group, mean pre-treatment serum AFP level was 71.07 ± 98.81 ng/mL (range: 1.3–356.0 ng/mL); mean pre-treatment tumor size was 39.45 ± 30.25 mm (range: 2.9–115.0 mm); mean post-treatment serum AFP level was 40.39 ± 74.99 ng/mL (range: 2.7–314.0 ng/mL); recurrence rate at 5 years was 62.3%; mean recurrent tumor size was 4.69 ± 4.65 mm (range: 4–13 mm); and mean survival time was 31.8 ± 28.57 months (range: 3–120 months) (Figure 1).

### TACE group

The TACE group included 27 patients (47.4% of the total), including 19 males (70.4%) and 8 females (29.6%) with a mean age of 73 ± 8.43 years (range: 40–86 years); 3 were in Child class A and 24 in Child class B. In the TACE group, mean pre-treatment serum AFP level was 345.58 ± 1175.90 ng/mL (range 2.2–6000 ng/mL); mean pre-treatment tumor size was 33.07 ± 18.02 mm (range: 10–76 mm); and mean post-treatment serum AFP level was 91.85 ± 143.75 ng/mL (range: 2.1–546 ng/mL). Twenty patients (74.07%) in this group underwent second TACE procedures because of tumor recurrence. The recurrence rate for the TACE group at 5 years was 92.2%. Mean recurrent tumor size was 25.00 ± 19.28 mm (range: 5–64 mm). Mean survival time was 17.2 ± 12.17 months (range: 1–50 months) (Figure 1).

### RFA group

The RFA group included 10 patients (17.5% of the total) including 3 females (33.3%) and 7 males (66.7%). Their mean age was 72.83 ± 7.01 years (range: 62–78 years); 2 patients had Child class A disease and 8 had Child class B disease.In the RFA group, mean pre-treatment serum AFP level was 130.45 ± 242.65 ng/mL (range: 2.3–779 ng/mL); mean pre-treatment tumor size was 29.42 ± 10.50 mm (range: 17–50 mm); mean post-treatment serum AFP level was 75.76 ± 137.99 ng/mL (range: 2.9–432 ng/mL); and mean survival time was 23.70 ± 21.87 months (range: 1–63 months) (Figure 1). Five patients (50%) in this group underwent second RFAs because of tumor recurrence. The recurrence rate for RFA patients at 5 years 73.5%. The mean recurrent tumor size was 25.92 ± 2.98 mm (range: 5–64 mm).

### Overall results

**Figure 2:**
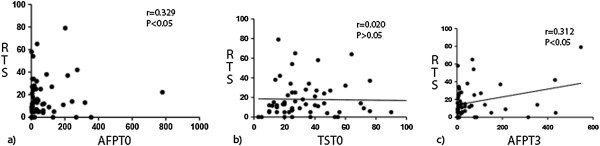
**Correlations between studied variables and recurrent tumor size in all patients. (a)** Pre-treatment serum AFP level (AFP_T0_) and recurrent tumor size (RTS) were significantly correlated (*r* = 0.329 *P* < 0.05). **(b)** Recurrent tumor size (TS_T0_) and RTS were not correlated (*r* = 0.020, *P* > 0.05). **(c)** The 3-month post-treatment serum level (AFT_T3_) and recurrent tumor size were significantly correlated (*r* = 0.312, *P* < 0.05).

The pre-treatment serum AFP level and tumor size, 3-month post-treatment serum AFP level, and time of any tumor recurrence were recorded. Statistical analyses showed significant correlations between pre-treatment serum AFP level (AFP_T0_) and recurrent tumor size (RTS) (*P* < 0.05) and between 3-month post-treatment AFP level (AFP_T3_) and RTS (*P* < 0.05). We saw no significant correlation between pre-treatment tumor size (TS_T0_) and RTS (Figure 2).

**Figure 3:**
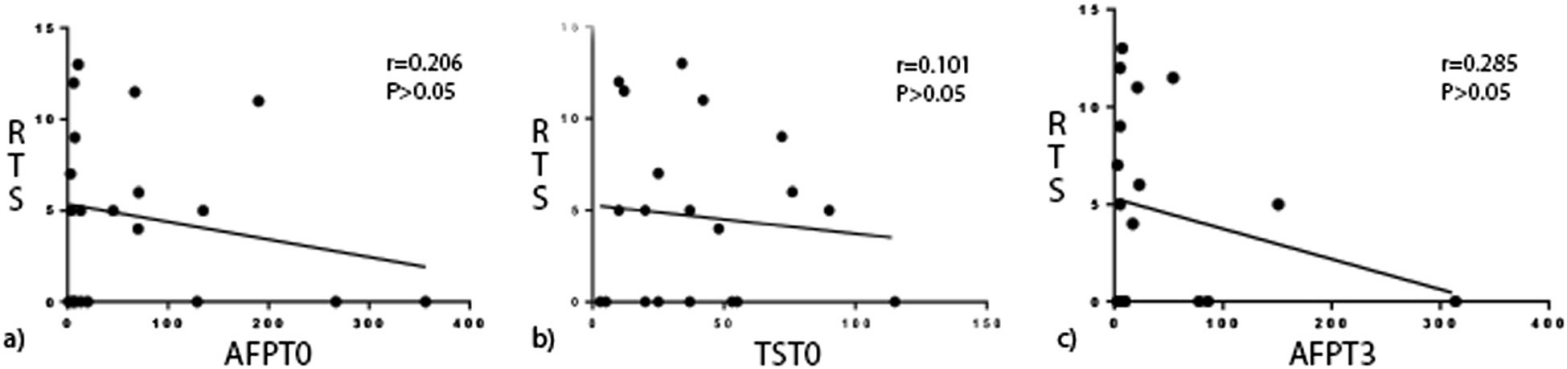
**Correlations between studied variables and recurrent tumor size in patients who underwent HR. (a)** Pre-treatment serum AFP level (AFP_T0_) and recurrent tumor size (RTS) were not correlated (*r* = 0.206, *P* > 0.05). **(b)** Recurrent tumor size (TS_T0_) and RTS were not correlated (*r* = 0.101, *P* > 0.05). **(c)** The 3-month post-treatment serum level (AFT_T3_) and RTS were not correlated (*r* = 0.251, *P* > 0.05).

**Figure 4:**
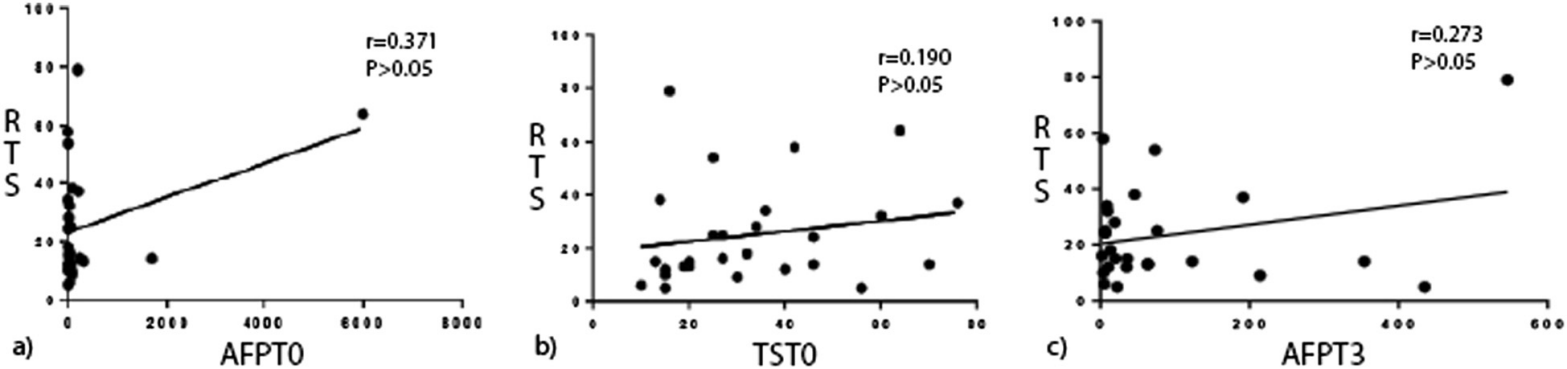
**Correlations between studied variables and recurrent tumor size in patients who underwent TACE. (a)** Pre-treatment serum AFP level (AFP_T0_) and recurrent tumor size (RTS) were not correlated (*r* = 0.371, *P* > 0.05). **(b)** Recurrent tumor size (TS_T0_) and RTS were not correlated (*r* = 0.190, *P* > 0.05). **(c)** The 3-month post-treatment serum level (AFT_T3_) and RTS were not correlated (*r* = 0.273, *P* > 0.05).

**Figure 5:**
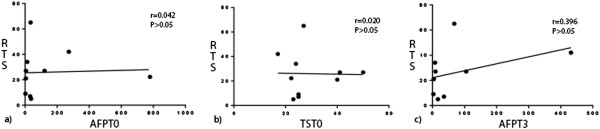
**Correlations between studied variables and recurrent tumor size in patients who underwent RFA. (a)** Pre-treatment serum AFP level (AFP_T0_) and recurrent tumor size (RTS) were not correlated (*r* = 0.042, *P* > 0.05). **(b)** Recurrent tumor size (TS_T0_) and RTS were not correlated (*r* = 0.020, *P* > 0.05). **(c)** The 3-month post-treatment serum level (AFT_T3_) and RTS were not correlated (*r* = 0.396, *P* > 0.05).

We saw no significant correlations between AFP_T0_, TS_T0_, or AFP_T3_ and RTS in the HR, TACE or RFA groups (Figures 3, 4 or 5, respectively), possibly because of the small numbers of patients in these groups. Comparisons of survival curves using the log-rank (Mantel–Cox) test showed that patients who underwent HR survived significantly longer than those who underwent TACE or RFA (both *P* < 0.05). Patients who underwent TACE and RFA did not significantly differ in survival times (Figure 1). Survival curves on χ^2^ analysis did not significantly differ.

## Discussion

HCC is one of the most common cancers worldwide, and is associated with a high mortality rate. Curative therapies such as HR or liver transplantation are feasible in 20–40% of patients [13]. TACE and RFA are the most commonly used treatment modalities when surgical excision is not indicated. In the present study, the rate of patients who underwent HR is higher than the rate reported in the literature. This is because only about half of the patients with HCC treated in our department over the study period were included in this study. If we consider all the excluded patients, their HR rate is quite similar to the rate reported in the literature [1,3,11].

Clinical studies suggest a relationship between serum AFP level and HCC severity. Serum AFP level may thus be useful for monitoring treatment response [14], and has been suggested to be directly related to tumor size. The ratio of serum AFP level to tumor diameter may be a better predictor of recurrence after curative resection than serum AFP level alone [10,15]. No published studies have investigated whether pre- and post-treatment serum AFP levels predict tumor recurrence size in patients who underwent HR, TACE, and RFA. In the present study, the variables and recurrent tumor size in the HR, TACE and RFA groups showed a correlation between pre- and post-treatment serum AFP levels and recurrent tumor size, whereas no correlation was found between pre-treatment tumor size and recurrent tumor size.

Several study groups recently proposed staging systems for HCC based on the extent of tumor and the underlying liver function, such as the Cancer of the Liver Italian Program (CLIP) score (based on tumor size), serum AFP level, Child-Pugh functional class, and portal vein thrombosis [16].

An average increase in serum AFP level of >7 ng/mL per month is a useful tool for diagnosing HCC in patients with liver cirrhosis and imaging findings that imply presence of tumors, especially when the serum AFP level is >200 ng/mL [2]. We have shown the usefulness of serum AFP level as a diagnostic tool. However, several factors limit its worth as a prognostic factor. Cut-off value is critical; a much higher cut-off value (600–1000 ng/mL) is required to predict prognosis compared with the diagnostic value (200–500 ng/mL). Extremely high serum AFP (>10,000 ng/mL) predicts poor outcome (3-year survival rate: 40%) compared with moderately elevated serum AFP (200–10 000 ng/mL; 3-year survival rate: ~70%) [10]. Serum AFP >1000 ng/mL is uncommon with tumors measuring <2 cm in diameter, whereas AFP may rise progressively to 1000–10,000 ng/mL as a tumor increases to >5 cm in diameter.

In contrast, Peng et al. [14] reported an association between mortality rate and serum AFP level. After 2 years, 130 of 160 patients (81.25%) in their study were still alive, with survival rates of 86.8% in patients with serum AFP level <20 _μg/L_, 88.9% with serum AFP level 20–250 _μg/L_, and 69.6% with serum AFP level >250 _μg/L_. These results show that HCC patients with a serum AFP level of >250 _μg/L_ had higher mortality rate than those with a serum AFP level of ≤250 _μg/L_. Although we did not use a specific cut-off limit in this investigation, patients with serum AFP levels above 250 _μg/L_ were considered to have poor prognoses.

The role of AFP in the genesis of HCC has also been studied. A retrospective study of 160 patients with HCC, of whom 72 received conservative treatment and 88 underwent surgical resection, and who were followed up every 6 months for 2 years, found that high serum AFP was associated with increased mortality in both treatment groups [14]. Apparently, AFP is not only a diagnostic marker, but is also a growth factor that promotes tumor progression, as supported by reports that higher serum AFP is associated with increased mortality [14]. AFP can promote HCC cell growth [17-19]. AFP^+^ HCC has higher cell proliferative activity than AFP^-^ HCC, as shown by the Ki-67 index [20], whereas AFP downregulation can suppress HCC cell growth [21,22].

The time from HCC resection to recurrence is an independent predictor of survival after recurrence [23,24]. Early recurrence (≤1 year after hepatectomy) has a worse prognosis than late recurrence (>1 year after hepatectomy) [25], due to the different pathogeneses of early and late recurrence. Large tumor size is also a risk factor for recurrence [25], but early recurrence is frequent even after curative resection of a small tumor [26]. Early recurrence after HR might result from intrahepatic metastasis from the primary tumor via the portal venous system, whereas late recurrence is more likely to result from multicentric tumor development. This might explain why some HCC patients have AFP^-^ primary tumors but AFP^+^ recurrent tumors: these tumors probably have different cell lines, which develop at different times. The current study found that the recurrent tumor size was correlated with 3-month post-treatment serum AFP level, and that survival was correlated with tumor size and serum AFP level.

We found a significant correlation between longer survival and low pre- and post-treatment serum AFP levels. We also found that patients who underwent HR survived longer than those who underwent TACE or RFA. When outcomes were analyzed within treatment groups, patients with low pre- and post-treatment serum AFP levels and small tumor size tended to survive longer, but not significantly so. This lack of significant correlations is probably because of the small numbers of patients in the treatment groups. However, the findings suggest that further study of these relationships is warranted.

HR is the treatment of choice for HCC patients without liver cirrhosis. Among patients with cirrhosis, candidates for HR should be carefully selected to reduce the risks of postoperative hepatic failure and death. The 5-year survival rate after HR can exceed 50% [5]. However, HR is associated with a high rate of local recurrence. In most cases, this is thought to result from occult micrometastases in the remaining liver parenchyma rather than inadequate surgical resection [27]. In the present study, long survival rates with low recurrence rates were due to the efforts to achieve safe oncologic margins during surgery.

A study of 12,118 HCC patients found that tumor diameter and serum AFP levels were predictive for patients who underwent HR, with tumors measuring < 20 mm in diameter having better prognoses than tumors measuring 20–50 mm in diameter [28]. This is consistent with our findings, in that patients who underwent HR with tumors <50 mm and lower pre-and post-treatment serum AFP levels had longer survival times.

The association of serum AFP level >200 ng/mL to increased risk of mortality was novel, but expected. Change in serum AFP levels (defined as a >50% decrease compared with baseline) after locoregional therapy are reportedly useful in assessing tumor response and survival [29], and for assessing lesions that have progressed on imaging examinations [30].

RFA is a popular local ablative therapy for unresectable HCC because of its safety and efficacy, but it is associated with a very high rate of recurrence (36%) [31]. Tumor diameter >2.5 cm is reportedly a risk factor for local recurrence [32], which may be related to the peritumoral vessels having a “heat-sink” effect on intrahepatic blood flow. RFA of large tumors can be technically challenging, requiring multiple overlapping spheres of tissue ablation under imaging guidance to ensure that no viable malignant tissue remains [33]. A histological study of patients who underwent liver transplantation after RFA found residual tumor tissue in 37% of patients with HCC ≤3 cm in diameter and 71% of patients with HCC >3 cm in diameter. Combining RFA with TACE can probably reduce the amount of such microscopic local tumor tissue [34].

A study that compared RFA and TACE in treating HCC within the Milan criteria found similar long-term survival rates in both treatment groups, but found that patients with smaller tumor volumes (<11 cm^3^) survived longer after RFA than after TACE [35]. However, we found no correlations between pre-treatment tumor size or serum AFP level and outcome in our RFA patients.

The current study evaluated relationships between treatment modalities and survival according to pre-treatment tumor size, but found no significant differences between patients who underwent TACE and those who underwent RFA. However, we found significant association between treatment modality and survival. Patients who underwent HR survived longer than those treated with TACE or RFA. Although international guidelines indicate RFA is more effective than surgery in selected patients, such as those with small tumors [5], we found no significant differences in survival between these two treatment modalities according to tumor size.

## Conclusions

Our results show that serum AFP levels are useful for diagnosing recurrence and predicting prognosis in HCC patients who have undergone HR, RFA or TACE. HR remains the treatment of choice for HCC in suitable patients.

## Competing interests

The authors declare that they have no competing interests.

## Authors’ contributions

AT and IDC made substantial contributions to conception and design; AA and MM acquired, analyzed and interpreted data; MCA and GM were involved in drafting the manuscript; HK and GB revised critical intellectual content; FP helped design the study and perform statistical analyses; and IDC gave final approval of the version to be published and agreed to be accountable for all aspects of the work in ensuring that questions related to the accuracy or integrity of any part of the work are appropriately investigated and resolved. All authors read and approved the final manuscript.

## Pre-publication history

The pre-publication history for this paper can be accessed here:

http://www.biomedcentral.com/1471-2482/14/40/prepub
